# Pituitary macroadenoma co-existent with supraclinoid internal carotid artery cerebral aneurysm: a case report and review of the literature

**DOI:** 10.4076/1757-1626-2-6459

**Published:** 2009-07-23

**Authors:** Chia-Sheng Wang, Tsung-Chih Yeh, Tai-Ching Wu, Chao-Hung Yeh

**Affiliations:** 1 Department of Neurosurgery, Chi-Mei Medical Center901 Chung Hwa Road, Yung Kang City, TainanTaiwan 710; 2 Department of Radiology, Chi-Mei Medical Center901 Chung Hwa Road, Yung Kang City, TainanTaiwan 710

## Abstract

With improved angiographic techniques and magnetic resonance angiography available today, an increasing number of incidental aneurysms are being detected. Occurrence of an intracranial aneurysm together with a pituitary adenoma presents tremendous risk to the patient, particularly when the aneurysm lies near the operative field.

A 61-year-old woman presented with a progressive visual field defect. Neurological examination revealed bi-temporal haemianopia. Cerebral magnetic resonance imaging and angiography revealed a pituitary macroadenoma co-existent with a cerebral aneurysm near the sellar region. The patient underwent an endovascular procedure for aneurysm embolisation and then underwent surgery for removal of the pituitary adenoma via a trans-sphenoidal approach.

We report our experience and emphasize the need for critical evaluation of neuroradiological examinations for precise diagnosis for avoiding a possible life-threatening situation.

## Introduction

The possible co-existence of an intracranial aneurysm and a pituitary adenoma has been previously reported in the literature [1,2,3]. With improved angiographic techniques and MR angiography available today, an increasing number of incidental aneurysms are being detected. Occurrence of an intracranial aneurysm together with a pituitary adenoma presents tremendous risk to the patient, particularly when the aneurysm lies near the operative field [4]. Because of invasion of the pituitary region, an intracranial aneurysm can sometimes mimic a sellar lesion. We report a 61-year-old woman with a pituitary macroadenoma co-existent with a cerebral aneurysm near the sellar region. Since such cases are possible life-threatening situations, the clinical presentation and management are discussed here.

## Case presentation

**Figure 1.**
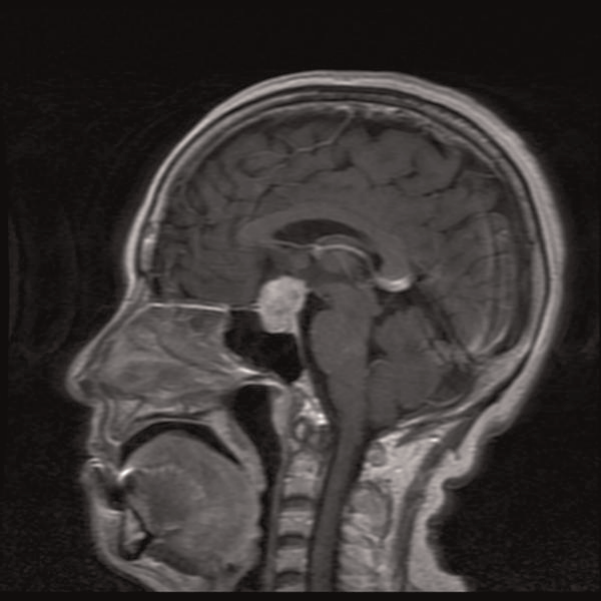
Gadolinium-enhanced sagittal MRI showing an expanding lesion inside the sella with suprasellar extension.

**Figure 2.**
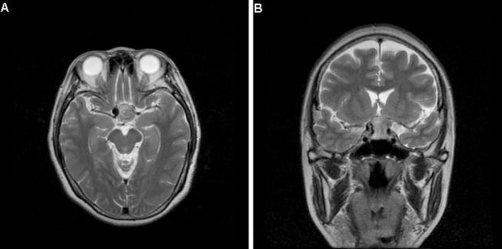
Axial **(A)** and Coronal **(B)** view of T2-weighted MRI showing a round flow-void mass in the right supraclinoid ICA associated with a pituitary macroadenoma.

**Figure 3.**
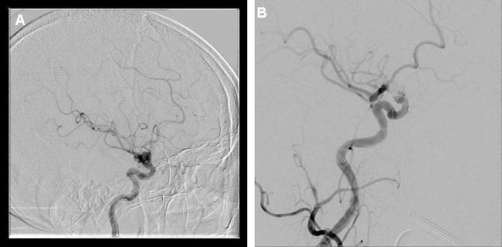
**(A)** Right internal carotid angiogram showing a wide-neck saccular-type aneurysm (measuring approximately 8 × 7.5 mm; neck, 4.8 mm) in the right supraclinoid ICA, pointing superiorly and with a small side lobe near its neck, pointing laterally. **(B)** Right internal carotid angiogram obtained after endovascular coil placement. The aneurysm has been completely obliterated.

A 61-year-old Asian Taiwanese woman presented to our institution with a progressive visual field defect. She had a history of hypertension for 20 years. Neurological examination revealed bi-temporal haemianopia. She also complained of occasional headache associated with nausea, vomiting and vertigo. Hormonal studies showed a slight elevation in prolactin levels. A brain computed tomography (CT) scan showed a slightly hyperdense lesion in the sellar region and a partially eroded sellar floor. Cerebral magnetic resonance imaging (MRI) revealed an expanding lesion inside the sella with suprasellar extension (Figure 1). On MRI, a flow void mass in the right supraclinoid internal carotid artery (ICA) raised the suspicion of a cerebral aneurysm (Figure 2). Cerebral angiography confirmed the presence of a wide-neck saccular-type aneurysm (measuring approximately 8.0 × 7.5 mm; neck, 4.8 mm) in the right supraclinoid ICA, pointing superiorly with a small side lobe near its neck which pointed laterally (Figure 3A). Initially, we treated the aneurysm by endovascular coil placement (Figure 3B). One month later, the patient underwent surgical decompression of the sella and excision of the tumour via a transnasal trans-sphenoidal endoscopic approach. Subtotal resection of the adenoma was performed, and the suprasellar portion was preserved to lower any possible risk to the coil-treated aneurysm. Histological examination confirmed the diagnosis of a prolactin-secreting adenoma. The patient’s post-operative course was uneventful, and the post-operative assessment of visual acuity, visual fields and extra-ocular movements showed no significant changes from the pre-operative assessment. The post-operative pituitary hormonal status was normal. The patient underwent adjuvant X-knife radiotherapy for the residual suprasellar portion of the adenoma. At 1-year follow-up, the patient had no neurological deficits, and MRI showed a normal pituitary gland and stalk.

## Discussion

In this report, a 61-year-old woman was diagnosed of a pituitary macroadenoma co-existent with a cerebral aneurysm near the sellar region.

Hypotheses for this phenomenon have been proposed, which include a direct mechanical effect of the pituitary adenoma on the vasculature [5], circulatory changes due to involvement of the skull base [6], direct infiltration by the tumour [7], and growth hormone production leading to arteriosclerotic and degenerative changes in the arterial walls of the circle of Willis [8,9], predisposing to aneurysm formation or enlargement.

The incidence of intracranial aneurysms in the general population is now considered to be around 5% [4,10-12]. The consensus is that the incidence of an intracranial aneurysm associated with pituitary adenoma is low. However, in the clinical series reported by Wakai et al. [9], the incidence of associated aneurysms with 95 pituitary tumours was 7.4%. In another retrospective study of 467 cases of pituitary adenomas, the incidence of associated intracranial aneurysms was 5.4% [4]. Thus, such association has been reported to range from 3.7% to 7.4% [4,13].

According to the previous review by Locatelli et al. [1], an intracranial aneurysm that co-exists with a sellar lesion is more frequently observed in patients with aneurysms of the ICA and the anterior communicating artery because they supply the pituitary region. In one retrospective study [4], 48% of intracranial aneurysms associated with pituitary adenoma were observed in the internal carotid-posterior cerebral artery followed by IC-ophthalmic aneurysms in 19% and IC bifurcation in 13%. This distribution pattern resembled that observed in other autopsy series of the normal population [14]. Moreover, 60% of aneurysms were near the parasellar region and 40% at distant locations [4].

Different clinical situations affecting the sellar region present with similar neuroradiological features. Pituitary tumour apoplexy, generally caused by acute expansion of an infracted or haemorrhagic pituitary adenoma extending laterally in the cavernous sinus or toward the optic chiasma and optic nerves, is a clinical situation characterized by a sudden onset of severe headaches associated with nausea, vertigo and/or altered mental status. Ophthalmoplegia and deterioration of visual acuity and visual fields are also frequently present. In cases of subarachnoid haemorrhage caused by the rupture of cerebral aneurysm, it may be sometimes difficult to differentiate it from pituitary apoplexy, particularly when the cerebral aneurysm lies near the sellar region. One case report described the thrombosis of a cerebral aneurysm of the intra-cavernous tract of the carotid artery mimicking apoplexy of a pituitary adenoma [1]; another study has reported the co-existence of aneurysmal subarachnoid haemorrhage with pituitary apoplexy [15].

Cerebral aneurysms can be treated by endovascular or microsurgical techniques [16-18]. In the present case, the patient experienced typical symptoms and signs due to the pituitary tumour and optic chiasma compression. With an aim to prevent the possible risk to the patient from the proximity of the aneurysm to the operative field, the cerebral aneurysm was first treated with endovascular coil placement; subsequently, surgical decompression of the sella was performed. The suprasellar portion of the pituitary adenoma was preserved during the trans-sphenoidal procedure. Our treatment plan in this case included initial endovascular embolisation for the cerebral aneurysm, followed by a transnasal trans-sphenoidal endoscopic approach for surgical decompression of the sella and excision of the pituitary adenoma below the diaphragm sella along with adjuvant X-knife radiotherapy for the suprasellar portion of adenoma-a plan that aimed to lower the possible risk to the patient during the interventional procedures.

Our experience reinforces the need for the critical evaluation of neuroradiological examinations. In such situations, the need for precise diagnosis is emphasized to avoid a possible life-threatening situation whenever an interventional approach is considered.

## Abbreviations

CT: Computed Tomography
ICA: Internal Carotid Artery
MRI: Magnetic Resonance Imaging
